# The effectiveness of a media-based intervention promoting help-seeking for mental health difficulties by Australian men: study protocol for a randomised controlled trial

**DOI:** 10.1186/s13063-022-06210-2

**Published:** 2022-04-11

**Authors:** Angela Nicholas, Simone Scotti Requena, Maria Ftanou, Simon Rice, Justine Fletcher, Andrew Mackinnon, Zac Seidler, Long Khanh-Dao Le, Cathrine Mihalopoulos, Jane Pirkis

**Affiliations:** 1 Centre for Mental Health, Melbourne School of Population and Global Health, The University of Melbourne, Parkville, Victoria 3010 Australia; 2 Orygen, Parkville, Melbourne, Australia; 3 Centre for Youth Mental Health, The University of Melbourne, Parkville, Australia; 4 Health Economics Division, School of Public Health and Preventive Medicine, Monash University, Melbourne, Victoria Australia

**Keywords:** Male, Media, Men, Help-seeking, Masculine, Masculinity, Mental health, Suicide, Suicide prevention, Video

## Abstract

**Background:**

Approximately one fifth of Australian males aged 16+ experience mood, anxiety or substance use disorders in a given year, and suicide by males accounts for three quarters of all suicides annually. However, males with mental health problems are less likely to seek and receive help than their female counterparts. Media campaigns with videos at their core are gaining popularity as a means of promoting help-seeking for mental health difficulties, but few studies have rigorously tested the impact of such videos. This randomised controlled trial tests the effectiveness of a short video promoting help-seeking by Australian men.

**Methods:**

This study is an online randomised controlled trial (RCT). Participants will attend an online group orientation session, where they will provide consent to participate and complete a baseline questionnaire (T1). After completion of the T1 questionnaire, participants will be randomised to watch either the intervention or control video on a 1:1 basis. After randomisation, participants will be able to access their allocated video for 1 week. Seven days after T1, participants will again be sent links to the video and to the post-exposure questionnaire (T2). One month after T2, participants will be emailed the follow-up questionnaire (T3). The primary outcome will be change in help-seeking intentions from T1 to T2. Secondary outcomes will be changes in help-seeking intentions from T1 to T3, changes in intentions to encourage other males to seek help, self-reliance, and male depression symptoms from T1 to T2 and from T1 to T3. The cost-effectiveness of the intervention will be evaluated. Participants will also complete questions about their opinions of the video and its effects at T2 and T3.

**Discussion:**

Our video-based intervention is designed to promote help-seeking for mental health difficulties among Australian men. If a lack of net harm is shown to be associated with viewing the intervention video, the video will be publicly released and could have broad impacts on individual and community attitudes towards help-seeking for mental health difficulties, and ultimately enhance men’s mental health and wellbeing. An evaluation of the effectiveness of the intervention is essential to ensure the intervention is achieving its objectives.

**Trial registration:**

anzctr.org.au 12621001008819

## Administrative information

**Table:** 

**Title**	The effectiveness of a media-based intervention promoting help-seeking for mental health difficulties by Australian men: Study protocol for a randomised controlled trial.
**Trial registration**	Australian New Zealand Clinical Trials Registry: 2621001008819
**Protocol version**	2 February, 2022, Version 1.2
**Funding**	Australian Government Department of Health - Medical Research Future Fund.
**Author details**	Centre for Mental Health, Melbourne School of Population and Global Health, The University of Melbourne; Orygen; Centre for Youth Mental Health, The University of Melbourne; Health Economics Division, School of Public Health and Preventive Medicine, Monash University.
**Name and contact information for the trial sponsor**	The University of Melbourne, Grattan Street, Parkville, Victoria, 3010.
**Role of sponsor**	The study researchers are employed at the University of Melbourne. Beyond the study researchers, the University has no role in the design or conduct of the randomized controlled trial (RCT). The Funder has no role in the design or conduct of the study.

## Background

Mental health problems are common in males, with 18% of Australian males aged over 16 experiencing mood disorders, anxiety disorders, and/or substance use disorders in a given year [1]. In Australia, the suicide rate for men is 18.6/100,000 population vs 5.7/100,000 for women [2]. On the whole, males with mental health problems and/or suicidal thoughts are less likely to seek and receive help than their female counterparts [3]. Men who adhere to traditional masculine norms are socialised in ways that reinforce rigid ideals of stoicism, independence, invulnerability, and avoidance of negative emotions [4]. A growing body of evidence demonstrates that conformity to these aspects of traditional masculinity may in part explain men’s poor mental health outcomes and their reluctance to seek help. For instance, conformity to traditional masculine norms has been associated with suicidality and suicide attempts [5, 6], depression in middle-aged and older males [4], alcohol and substance use [7], and negative attitudes towards help-seeking [8].

Media campaigns are one approach being used to promote help-seeking for mental health difficulties at a population level. Media campaigns have historically used a range of media, such as television and radio community service announcements, posters, and billboards to promote help-seeking messages [9, 10]. Although such campaigns are gaining popularity, and despite widespread and ongoing investment in such campaigns, rigorous examination of their effects is still relatively limited [9, 10]. These campaigns may be having no effect or, although unlikely, it may be that they are doing harm in some way. Our randomised controlled trial (RCT) testing the effects of a 3-h documentary relating to suicide among Australian men (‘Man Up’) showed a significant increase in intentions to seek help and to encourage others to seek help, as well as a significant reduction in adherence to traditional masculine norms [11]. The results of this RCT show that a video-based intervention aimed at encouraging help-seeking among men can be effective in promoting help-seeking.

However, a 3-h documentary requires significant investment both in terms of production costs and viewing time. Through this RCT, we will test whether a similar message to that promoted in the 3-h ‘Man Up’ documentary, encouraging men to seek help when they are experiencing mental health difficulties, can be effective when delivered through a much shorter video. In this RCT, we will test whether a 4-min video is similarly effective in promoting help-seeking among Australian men. If this video is shown to have no net negative effect on help-seeking, this video will be publicly released. The release of the video will be supported through a comprehensive media campaign aimed at promoting viewing of the video and for viewers to visit a purpose-designed website, which will house help-seeking and help-offering information, as well as other supporting videos and content.

Data assessing men’s help-seeking intentions, intentions to encourage other men to seek help, self-reliance, depression symptoms, and opinions regarding the allocated video will be collected across three timepoints: baseline (T1), post-intervention (T2—1 week after baseline), and 4-week follow-up (T3—4 weeks after T2). Participants will view the video between T1 and T2.

## Study objectives

### Primary objective

To assess whether a novel, 4-min intervention video increases men’s help-seeking intentions from baseline (T1) to post-intervention (T2).

### Secondary objectives

To evaluate the effectiveness of the intervention on increasing help-seeking intentions and intentions to encourage other males to seek help from baseline (T1) to follow-up (T3) and to evaluate the effectiveness of the intervention on reducing scores on the masculine norm of self-reliance from T1 to T2 and T1 to T3, and male depression symptoms from T1 to T3. We also aim to evaluate the cost-effectiveness of the intervention if it is shown to have a significant positive effect on help-seeking intentions.

## Methods

### Study design

This is a single-blinded, two-arm RCT comparing the effects of an intervention video against a control video. We plan to recruit 446 adult Australian men to be randomised, with a 1:1 allocation (223 participants per arm).

### Study setting

This study will be conducted entirely online. Participants will be recruited across Australia, with no geographical restrictions. They will attend an orientation session with up to nine other participants via the online web conferencing software, Zoom.

### Participant selection

When participants register their initial interest in participating in the RCT via a registration website, they will be sent a detailed Plain Language Statement (PLS) outlining the requirements of trial participation. They must then attend an online orientation session run by one of our researchers and a study psychologist. In these sessions, the researcher will reiterate key background information from the PLS and answer questions to allow participants to provide informed consent. Once participants have had their questions answered, they will complete an online consent form, ticking a box to provide consent to continue to the T1 questionnaire (note that this includes consent to use of de-identified data for additional studies with ethical approval). If they do not consent, they will not proceed to the T1 questionnaire. The Depression Symptom Inventory—Suicidality Subscale (DSI-SS) [12] suicide risk screening questionnaire will be included in the T1 questionnaire. The study psychologist who attends the session will phone any participant who scores 2 or above on the DSI-SS to further assess their risk and to assist the participant in seeking further mental health support if the participant would like it. If the psychologist determines that the participant is at imminent risk of suicide or could be harmed by further participation, they will be excluded from further participation and referred for mental health support. If the participant is not determined to be at risk of harm, they will continue in the trial, but may still be referred for further support as required. The study psychologist will conduct a risk assessment using criteria such as whether the person has a current plan for suicide, and whether they have a history of suicide attempt. Participants will also be supplied with the psychologist’s contact details and able to contact them directly for further support for up to 4 weeks following completion of the T3 questionnaire. Following completion of the T1 questionnaire, participants will be randomised to view the intervention or control video.

#### Inclusion criteria

- Aged 18 years or over
- Identifies as male
- Resides in Australia
- Understands and reads English

#### Exclusion criteria

- Scored ≥ 2 on the DSI-SS [12], and the study psychologist determines that the participant is actively suicidal or likely to experience harm from further participation.

### Procedure

#### Recruitment

Recruitment will occur online. To ensure participation from a broad cross-section of Australian men, we will use a variety of online recruitment methods. We will use our project partners (e.g. Gotcha4Life, a men’s suicide prevention organisation), and personal and professional networks to recruit participants, as well as public channels. We will send a recruitment flyer via email to researchers providing advice to the project, partners and own networks requesting they in turn share the recruitment information with their networks and via their mailing lists, websites and social media accounts, including Twitter, Instagram, Facebook and LinkedIn. We will post the recruitment information on the University of Melbourne student portal and in student newsletters aimed at departments with significant male enrolments such as Engineering, and Business and Commerce. We will also encourage snowballing (for those who participate to also recruit others).

Participants will be informed via the recruitment materials that the study relates to the attitudes of Australian men to mental health and wellbeing. We have chosen to present this aim of the study as it accurately orients participants to the context of the study without creating unnecessary bias by stating more specifically that we are examining the effects of a video on help-seeking intentions for mental health difficulties.

#### Assignment of interventions

##### Treatment allocation and concealment

All participant data will be collected and managed by an external data management contractor, Strategic Data Pty Ltd (now trading as Logicly). Allocation will be conducted using computer-generated random numbers using an automated data management system after completion of the T1 questionnaire. No stratification will be used.

The researchers, including the statistician responsible for the analysis of outcomes, will be blinded until after the completion of analysis to assess the primary outcome (comparing T1 and T2 data), which will take place after completion of T2. The study psychologist will also be unaware of any participant’s allocation unless they describe the video they have seen in the course of their interaction. If this were to occur, the psychologist would not reveal the participant’s allocation to the researchers.

Because they are aware of the content of the video they watch, it will not be possible to totally blind participants to their allocation. However, they will not be made aware that they could have been allocated to watch either an intervention or control video until T3. In the T3 questionnaire, we will explain the existence of the intervention and control group and ask participants to indicate the group to which they believe they have been assigned. Therefore, we will attempt to maintain the blinding of participants to the degree possible within this study design.

##### Intervention

The intervention is a music video of approximately 4-min length created specifically for this study. It features a large group of diverse men singing a song in a community town hall. The song’s lyrics relate to the need for men to talk to others when experiencing mental health difficulties and to encourage others to do the same. The ‘call to action’ at the end of the video is ‘When the going gets tough. Get Talking’, and then there is a link to a website, which will provide links to further mental health support. This website will not be live during the trial to ensure that we can isolate any effects of the video from any effects of additional actions that might take place after participants watch the video.

##### Control

The control video is a 4-min section of a longer documentary video that relates to improving cognitive processing speed by playing table tennis. We chose this video as it is unlikely to influence the outcomes of interest in the study, and as it relates to brain health, though not mental health, and the study questionnaires will seem relevant to the video. The control video also shares some common features with the intervention video, including that it is the same length, is also Australian, and features only men.

There will be no special criteria for discontinuing or modifying allocated interventions.

#### Treatment adherence

On day 3 after T1, participants will be encouraged via an email reminder including a personalised video link to watch the video. These links will provide participants with unlimited access to their allocated video until the completion of the T2 questionnaire. Participants will be sent the video link again with the link to complete the T2 questionnaire at day 7 after T1. To assess adherence to the intervention, we will record participants’ viewing behaviour related to the intervention and control videos by counting how many times the given video was started, how many times it was watched from beginning to end, when the video was paused, the number of times the participant tried to navigate away from the video page (e.g. by closing the tab or browser) and at what time points in the video the participant left the video page (e.g. 14 s). We will also ask one ‘attention’ question related to the content of the intervention or control video at T2. This is a question the participant can only answer by having attentively watched the video.

#### Relevant concomitant care permitted or prohibited during the trial

Participation in the trial does not require any alteration to participants’ usual care pathways (including use of any medication) in either arm.

#### Provisions for post-trial care

All participants will have free access to short phone consultations with the study psychologist throughout the trial and for 4 weeks following their completion of the trial. The psychologist will refer them to further supports as necessary.

### Participant timeline

**Fig. 1:**
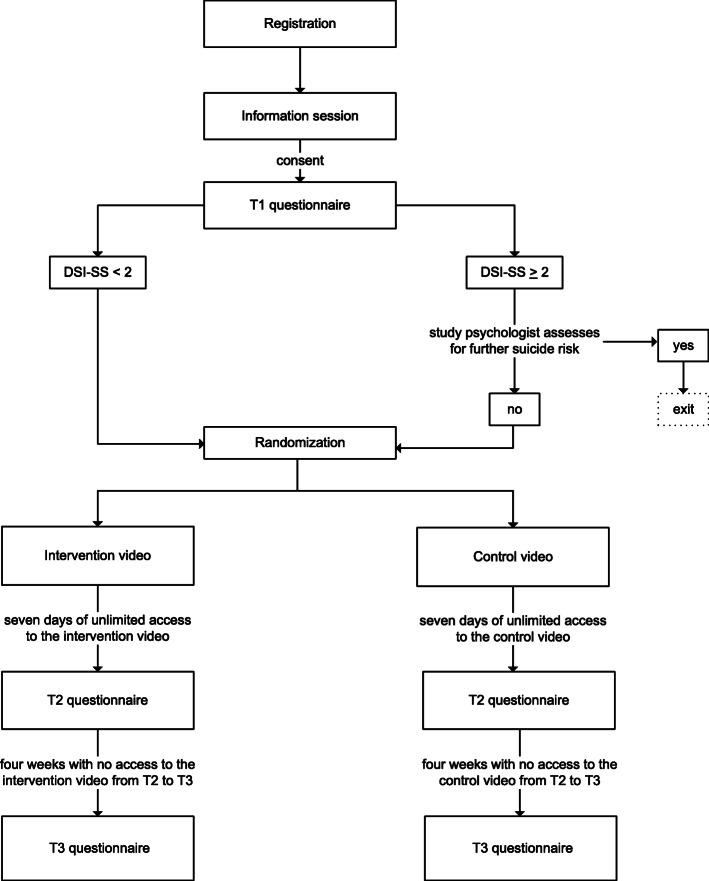
Timeline for the trial. *Note:* DSI-SS, Depression Symptom Inventory—Suicidality Subscale [12]. See Table 1 for further information about T1, T2, and T3 questionnaires and timing

After registering their interest in participation, participants will attend an online information session. After consenting, participants will immediately be given a link to complete the T1 questionnaire, which will include questions about socio-demographics, suicide risk, help-seeking intentions, intentions to encourage other males to seek help, male depression symptoms, self-reliance, health service use, and social support. Following completion of the T1 questionnaire, participants will be randomly assigned to intervention or control groups by the independent data management contractor. Immediately after randomisation, Strategic Data will email participants a link to their allocated online video (intervention or control); they will also receive a reminder to watch the video at day 3. They will be sent the video link again on day 7, along with the link to the T2 questionnaire. The T2 questionnaire will include questions about help-seeking intentions, intentions to encourage other males to seek help, self-reliance, social support, and feedback on the video. If participants do not view the video within 1 week of receiving the link or do not complete the T1 questionnaire, two reminder emails will be sent 10 and 12 days after T1. The link will expire after 1 week and participants will no longer be able to complete the T2 questionnaire. One month after T2, participants will be emailed the T3 questionnaire, which replicates the T1 questionnaire with the exceptions that it also includes questions about the effects of viewing the allocated video and does not include the DSI-SS. Participants will receive two reminders 3 and 5 days after the initial T3 email is sent. The link will expire after 1 week and participants will no longer be able to complete the T3 questionnaire. All participants who complete the T1 questionnaire will proceed to the T3 questionnaire, regardless of whether they complete the T2 questionnaire or watch their allocated video. See Fig. 1 for the timeline for the trial and Table 1 for the schedule of assessments.

**Table 1:** Schedule of assessments

Variable	Measure	Timeline		
		T1	T2	T3
*Screening measure*				
Suicide risk	Depression Symptom Inventory—Suicidality Subscale [12]	X		
*Primary outcome measure*				
Help-seeking intentions	General Help-Seeking Questionnaire—Self [13]	X	X	X
*Secondary outcome measures*				
Intentions to encourage other males to seek help	General Help-Seeking Questionnaire—Other [13]	X	X	X
Self-reliance	Conformity to Masculine Norms Inventory 30—Self-reliance subscale [14]	X	X	X
Male depression symptoms	Male Depression Risk Scale-7 [15]	X		X
Health service use	Health Service Use Questionnaire	X		X
*Other measures*				
Socio-demographics	Information about age, postcode, country of birth, language, marital status, current student, highest education, and employment	X		
Social support question	Information about the number of close friends and close relatives	X	X	X
Feedback on video	Information about video enjoyment, likeability, take-home message, degree of intended sharing with others, and belief about impact		X	
Effects of viewing the video	Information about life changes and time spent with friends			X

*Note.* T1, the T1 questionnaire is completed in the online information session; T2, the T2 questionnaire becomes available 7 days after completion of the T1 questionnaire and the survey expires after 1 week; T3, the T3 questionnaire is made available 4 weeks after the T2 questionnaire is completed or expires. The survey link expires after 1 week

### Measures

#### Screening measure

The Depression Symptom Inventory—Suicidality Subscale (DSI-SS) is a 4-item self-report measure of suicide risk [12]. Scoring 2 or above on the DSI-SS will trigger a follow-up call from a study psychologist. Examples items include ‘Sometimes I have thoughts of killing myself’ (score = 1) and ‘In some situations, I have impulses to kill myself’ (score = 1). The DSI-SS has demonstrated good internal consistency; convergent validity with a range of measures, including hopelessness and lack of interpersonal belonging; and the ability to differentiate between people with and without suicide attempts [15]. Despite the established cut-point for suicide risk in the general population being 3, we have chosen 2 as the cut-off for follow-up assessment to minimise risk to participants.

#### Primary outcome measure

The General Help-Seeking Questionnaire—Self (GHSQ-Self) is a 13-item self-report measure of help-seeking intentions [13]. The GHSQ is a valid and reliable measure of intentions to seek help when experiencing personal difficulties [13]. The GHSQ-Self asks, ‘If you were having a personal or emotional problem, how likely is it that you would seek help from the following people or services?’ Ten responses are provided: *intimate partner*, *friend*, *parent*, *other family members*, *mental health professional*, *phone helpline*, *doctor*, *minister or religious leader*, *I would not seek help from anyone*, and *I would seek help from another not listed above*. We have modified this scale by providing an additional three responses: *online health chat rooms*, *online searches for health information*, and *social media*. Respondents rate the likelihood on a 7-point scale (1 = *extremely unlikely* to 7 = *extremely likely*); scores are summed, and a higher total score indicates greater intentions to seek help. The GHSQ is widely used and was used in our previous RCT of ‘Man Up’, discussed in the ‘Background’ section of this paper [11].

#### Secondary outcome measures

The General Help-Seeking Questionnaire—Other male (GHSQ-Other) is an adapted version of the GHSQ [13], and is a 13-item self-report measure of intentions to encourage other males to seek help. The GHSQ-Other format is as the GHSQ-Self above, with the variation that the question prompt asks: ‘If a male friend or family member of yours was having a personal or emotional problem, how likely is it that you would recommend to that person to seek help from the following people?’

The Conformity to Masculine Norms Inventory-30—Self-Reliance subscale (CMNI-30-SR) is a 3-item self-report measure of self-reliance [14]. The CMNI-30 assesses conformity to traditional masculine norms across 11 norms, including self-reliance. The self-reliance subscale relates to willingness to ask for help when needed. Participants rate their agreement (1 = *strongly disagree* to 3 = *strongly agree*) with three responses: ‘It bothers me when I have to ask for help’, ‘I never ask for help’, and ‘I am not ashamed to ask for help’. The subscale score is a sum of responses, with higher scores indicating higher levels of self-reliance.

The Male Depression Risk Scale-7 (MDRS-7) is a 7-item self-report measure of male-specific depression symptoms [15]. It assesses symptoms in six domains: emotion suppression, drug use, alcohol use, anger and aggression, somatic symptoms, and risk-taking. Each item is rated for how frequently the respondent has experienced that symptom over the last month (0 = *none of the time* to 3 = *all of the time*). Items can be summed to provide an overall depression score.

The Health Service Use Questionnaire (HSUQ) is a modified version of the self-report measure of the Resource Use Questionnaire [16] developed for use in this study. The HSUQ has eight items measuring health service use. These eight items will be used to assess participants’ use of a range of health services for mental health difficulties over the past 4 weeks. Use of a range of services is measured, including primary health services (e.g. consultations with a general practitioner, psychologist), inpatient admissions (e.g. hospital, community care unit), Internet-based services (e.g. information pages, online support group), and phone counselling. Use of psychotropic medications (e.g. sleeping tablets, antidepressants) is also assessed, as is the effect of mental health difficulties on functioning at work/in education.

#### Other measures

Social support will be measured by a single item measure, which reads: ‘About how many close friends and close relatives do you have (people you feel at ease with and can talk to about what is on your mind)?’ This item will be used in analyses using scores from the GHSQ-Self, to account for the possibility that low intentions to seek help may be related to a lack of availability of friends or relatives from whom participants can gain support.

At T2, we will ask participants a multiple-choice knowledge question about the content of the video to assess their attention to the video; we will ask them to rate from 1 (not at all) to 10 (very much) how much they enjoyed the video; and two open-ended questions about the parts they liked most and least; one open-ended question about the take-home message of the video. We will also ask them to indicate on a scale from 1 (no impact) to 10 (a great impact) the impact the video would have on men’s health and wellbeing.

At T3, we will ask participants different questions about the effects of the video. We will ask ten questions regarding whether the video has resulted in their changing their behaviour (e.g. spending more time with male friends), the parts of the video that had the most positive and negative impacts and their effects, whether they talked to others about the video and their likelihood of liking or sharing the video online. Some of these questions are closed ended (i.e. Yes/No) and some use rating scales (e.g. 1 (No, not at all) to 10 (Yes, a lot)). Others ask for explanatory free-text comments (e.g. Now tell us in your own words what were the parts of the video, if any, that had the most positive impact on you and explain why). We will also ask whether the participant believes they were allocated to the intervention or control group, after a brief explanation of the meaning of these conditions.

A copy of the full set of measures can be supplied upon reasonable request to the corresponding author.

### Participant retention and follow-up

Researchers will follow up twice by phone and/or email with participants who register to attend an orientation session, but do not attend, in order to encourage their participation in the trial. Participants will be sent two email reminders to complete the T2 and T3 questionnaires within 1 week of being sent these questionnaire links. The questionnaire links expire after 1 week. Participants will be provided with online vouchers as reimbursement for their time at the completion of each data collection point: T1 = AUD30; T2 = AUD30; T3 = AUD40; Total = AUD100.

### Sample size and power analysis

The sample size is calculated to detect a 0.25 standardised difference in mean on the GHSQ-Self score between the intervention and control group (90% power, 2-sided 5% significance level), assuming a correlation of 0.67 between T1 and T2 scores. This sample size of 446 participants also allows for 20% loss to follow-up. We have chosen a relatively small effect size given the universal nature of the intervention, where we would aim to create change by producing a small effect on a large number of men, and given that we are measuring intentions to seek help as the primary outcome, rather than actual help-seeking behaviour. Intentions was chosen over behaviour as the primary outcome given the small amount of time between T1 and T2 in which participants would have the opportunity to seek help as the need arose.

### Data collection and management

Data will be collected via an online system administered by the external data management company, Strategic Data Ltd. Data will be entered directly by participants into the online questionnaires, minimising the chances of missing data and data entry mistakes. With the exception of some free-text response questions, questionnaire responses will be restricted to those values that are valid responses for each question (e.g. maximum 28 days if questions ask ‘on how many days in the last four weeks…’). Warning messages will be triggered if invalid entries are entered or if participants have not answered mandatory questions.

Strategic Data will set up and host the online forms and technology to collect the participant data. The hosting is in a highly secure Australian-based environment. The participant’s access is password protected and locked upon completion of each survey. This means that if anyone accesses the participant’s username and password once their participation has ended, their record will not be accessible. This further will further minimise the risk of inappropriate access.

Once the data collection is complete and upon request from one of the two lead investigators, Strategic Data will supply a final encrypted version of the dataset for 5-year storage at University. Respondent questionnaire data and identifying information will be sent separately and will be encrypted and password-protected. Strategic Data will then delete all respondent data from its active systems, leaving only backups which will be deleted over time in the normal course of backup protocols.

The trial manager will manage the data at the University of Melbourne. Online survey data will be password-protected, and exported data files will be stored on the university server in a password-protected folder to which only the researchers employed on this trial will have access. Upon completion of the trial, all data and related information will be stored on the secure university server. This information will not be deleted until any continued interest in the information ceases or until at least 5 years following the final publication relating to that research.

### Confidentiality

Strategic Data will have participants’ identifying data for the purpose of sending personalised email invitations and reminders. They will match the data over the three data collection points by allocating participants a unique identifier and will store identifying information separately from the questionnaire data. The only time participants’ identifying data and questionnaire data will be linked will be if the participant scores above the cut-off for suicide risk. In this instance, Strategic Data will supply the researchers and the study psychologist the following data for the purpose of following up the participant: the participants’ name and phone number, the name and contact number of their nominated emergency contact person, their total score on the screening questionnaire (DSI-SS), and their responses to the DSI-SS items. At the end of the data collection period, Strategic Data will supply our research team with both the database containing both the participants’ details and another database containing the questionnaire data. These will be sent and stored separately. These databases will then be stored on the University’s secure server.

### Statistical analyses

#### Primary outcomes

Analyses of continuous outcomes will be undertaken on an intent-to-treat basis, including all participants who have been randomised, regardless of their actual receipt of the intervention or withdrawal from the study. Mixed-model repeated measures (MMRM) analyses will be used because of the ability of this approach to include participants with missing data. The model will include factors of intervention arm, occasion of measurement (T1, T2), and their interaction. The effectiveness of the intervention will be assessed by a planned comparison of the difference between groups in change of the primary outcome variable from T1 to T2. An unstructured residual variance-covariance matrix will accommodate within-participant dependency. Tests of significance will use degrees of freedom adjusted using the Kenward-Roger method based on the observed information matrix. If necessary, transformation of the outcome variable will be undertaken to ensure distributional assumptions of the model are met.

Analysis of primary outcomes and some secondary outcomes will take place when all T2 post-intervention data have been collected, but before T3 follow-up data have been collected. This analysis will assess the effectiveness of the intervention from T1 to T2 on increasing participants’ intentions to seek help for mental health difficulties (primary outcome), and intentions to encourage other men to seek help and self-reliance (secondary outcomes).

At this time, we will assess whether there is evidence of a lack of net harm associated with viewing the intervention video. Doing this will allow us to inform the production company tasked with releasing the video into the public domain and promoting public access to the video whether they can release the film in the following month. This release will be dependent on the analysis of primary outcomes showing evidence of lack of net harm. Lack of net harm will be indicated by the lower confidence interval of the mean GHSQ-Self score in the intervention condition post intervention being no lower than that in the control condition, adjusting for any baseline differences. The parameters will be estimated within the framework of the models used to assess the effectiveness of the intervention (i.e. we will use an MMRM to obtain the estimated means and their confidence intervals).

We have chosen the change in help-seeking intentions between T1 and T2 as our primary outcome for two main reasons. First, we expect that if there is an effect of this short intervention video, it will be immediate. We are encouraging men to seek help for mental health difficulties before they escalate. These sorts of difficulties are extremely common, and the help-seeking message is therefore likely to be immediately salient to most participants. We recognise that while intentions to seek help can change immediately, actual help-seeking may take longer. Therefore, we will assess help-seeking behaviour as a secondary outcome at T3 (1-month after baseline), as well as whether any increase in help-seeking intentions has been retained. Second, in the context of our collaboration with our film and advertising colleagues, we have created a broader campaign within which this video is at the core. The broad campaign will promote the video within the context of a range of other campaign materials promoting help-seeking. Within this trial, we want to be sure that the video does no harm to participants and ideally has a positive impact, but we also anticipate that by itself, this short video would be likely to have a short, sharp impact, while the broader campaign will have a greater, more lasting impact. We will do further empirical evaluation to test the impact of the broader campaign once it has been released. Therefore, the findings of this trial form a broader assessment of the campaign impacts that focus purely on the shorter-term effects of the video viewing.

#### Secondary outcomes

Analyses of other outcome variables will follow the same methods as the primary outcome. Secondary outcomes will also include change in the primary and other outcome variables from baseline (T1) to follow-up (T3).

#### Subsidiary analyses

Subsidiary analyses will be undertaken to examine potential moderators of effectiveness and outcomes in subgroups. These subgroups will be defined by age group, English spoken at home vs other language spoken at home, and number of viewings of the video. These analyses will add the potential moderator variable as a factor to the model used in the main analyses. Incremental models will evaluate its significance as a stable and as a time-varying covariate and fit a three-way interaction of the moderator with group assignment and occasion of measurement. Comparisons within the latter interaction will address whether change over time differs significantly between intervention arms within subgroups and whether such differential changes differ significantly between subgroups. Additional exploratory analyses may be conducted with additional variables including self-reliance scores on the CMNI-30-SR and depression scores on the MDRS-7.

#### Cost-effectiveness analysis

In subsequent analyses, a trial-based economic evaluation will be conducted based on the help-seeking intention scores, the intervention costs, and mental health-related services costs. Intervention costs include the intervention development costs (e.g. planning, development, and production stages of video). Mental health services cost will be calculated by applying standard Australian unit costs (i.e. Independent Hospital Pricing Authority [17], Medicare Benefit Scheme fees [18]) to the resource use units collected through the HSUQ. Mean values of costs and help-seeking intention scores will be reported for both groups and assessed by generalised linear models. An incremental cost-effectiveness ratio (ICER) will be calculated as the difference in average cost between the groups, divided by the difference in help-seeking intention scores. Nonparametric bootstrapping will be used to obtain confidence intervals for incremental cost-effectiveness ratios. A modelled economic evaluation will also be undertaken using the results of this trial and relevant epidemiological literature to extrapolate long-term costs and consequences associated with help-seeking intentions.

#### Plans to give access to the full protocol, participant-level data, and statistical code

The datasets analysed and statistical code used for analysis may be made available from the corresponding author on reasonable request.

## Oversight and monitoring

### Composition of the coordinating centre and trial steering committee

The Centre for Mental Health, Melbourne School of Population and Global Health, at the University of Melbourne, will be the trial coordinating centre. Staffing from the centre will consist of a trial manager (AN), who will be responsible for ensuring that the research team, study psychologists, and data management company (Strategic Data Ltd) conduct the trial, and that data is managed, in accordance with the protocol. There will be three additional internal researchers responsible for data collection (MF, SSR, JF). These researchers will meet weekly with the trial manager and will have other contacts as needed throughout the data collection phase. The two chief investigators (JP, SR) with have oversight of the project, with statistical and health economics advice and analyses provided by external experts (AM, LL, CM). In the months leading up to the trial, the chief investigators will meet weekly with the research team, including the trial manager and one external researcher with expertise in suicide prevention for men (ZS), to ensure all systems are in place to adhere to the protocol. Throughout the data collection phase of the trial (6 weeks), this group will meet on an as-needed basis, with daily contact between the internal chief investigator (JP) and the trial manager to ensure adherence to the protocol. This trial is one of seven RCTs that form part of a larger research programme (the Buoy Project). The chief investigators from these seven trials, and the trial manager from this trial, will meet monthly to receive trial updates and to discuss trial-related issues. This group of experienced researchers will act as the steering committee to this trial and can also provide ad hoc input to the trial as requested.

Given the relatively low-risk nature of the trial, a separate data monitoring committee will not be established.

### Adverse event reporting and harms

This is a low-risk intervention, and no significant adverse events or severe adverse events are anticipated. No adverse events were reported for the trial on which the methods are based [11], despite that trial testing a much longer (3-h) and suicide-specific video intervention. The most likely adverse event would be some increase in distress related to viewing the video, which has indirect references to suicide. We will provide the contact details of a study psychologist to all participants and encourage them to contact the psychologist should they experience any distress during and in the 4 weeks following participation in the trial. Any reported severe adverse events would be reported to the Human Research Ethics Committee that approved this protocol and advice sought from them regarding the actions required to respond to the specific event.

### Plans for communicating important protocol amendments to relevant parties

Any deviations from the protocol will be documented by the trial manager and signed off by the chief investigators. This document will then be stored by the trial manager with all other trial documentation. In addition, deviations from the protocol likely to have ethical implications will be reported to the Human Research Ethics Committee and advice will be sought on the necessary response to the deviation. The trial manager will also be responsible for updating the relevant entry in the Australian New Zealand Clinical Trials Registry (No. 12621001008819).

## Dissemination

We will prepare a lay summary of the outcomes of the RCT and will email it to all participants who indicate on the consent form that they are interested in receiving these results. We will publish several peer-reviewed journal articles and deliver presentations at national and international conferences. We will also deliver other presentations about the trial to other mental health and suicide prevention researchers and professionals. If the intervention video is shown to have no net negative effect on help-seeking, it will be publicly released and its release will be supported by a media campaign that will include the video on a project website with complementary content, social media dissemination, media interviews, and other strategies for increasing public interaction with the video.

## Discussion

This RCT seeks to test the effectiveness of a 4-min intervention video aimed at improving help-seeking for mental health difficulties among Australian men. The intervention video tested in this trial will form part of a larger media campaign aimed at improving help-seeking for mental health difficulties among Australian men.

This RCT will be conducted completely online. Our research team has designed this online trial to ensure it can be completed in the current Australian environment in which much of the population is in ‘lockdown’ due to the Covid-19 pandemic, prohibiting face-to-face interactions with participants and travel for data collection. Our methodology could be adopted for similar purposes with other interventions, but also applied to the development of media campaigns aimed at improving mental health outcomes in other countries or targeting other population groups (e.g. older people or those living in rural communities). Although our research team has conducted similar RCTs with media interventions, this is the first to make use of an online group orientation meeting. Securing the attendance of potential participants at this meeting is a potential challenge, as are the logistics of running such a group session online. However, with current (and possible future) insecurities around in-person group gatherings due to the risks posed by Covid-19, it is a useful RCT design and one that could be capitalised on in the future.

We recognise a number of potential biases to be introduced within this trial. While the RCT design attempts to control a number of these, we have also taken additional steps beyond the randomised double-blind design. One of the most likely biases is in participant selection. In our recruitment advertising, we have attempted to minimise the amount of specific information provided on the purpose of the study while providing enough information to allow participants to provide informed consent to participate. To wit, we have stated in the recruitment materials that the study is about men’s attitudes towards mental health and wellbeing. We recognise that this advertising is most likely to attract those with a particular interest in mental health who may not represent the broader population. However, we will attempt to minimise this selection bias by advertising through a range of channels that target men who do not necessarily have a high level of mental health literacy. For example, we will target university students studying in disciplines such as business and engineering rather than targeting those in the health or social sciences. We will also target a wide range of groups such as parenting and sporting groups and the networks of our film and advertising colleagues, who might have less exposure to mental health information than our networks.

We also recognise that, given the participants will meet at least one researcher in the online baseline data collection session, there is potential for social desirability bias. That is, that participants may be inclined to respond in the way they expect the researchers would prefer. We would expect this effect to be equal between intervention and control arms, particularly as the researchers are blind to participant allocation, but we have also chosen to use for the assessment of our primary outcome a change-in-change analysis. We will assess whether the intervention group shows a greater increase in help-seeking intentions than the control group, rather than a specified level of change within groups, thereby controlling for a consistent level of social desirability bias between the groups.

## Trial status

This RCT has been the Australian New Zealand Clinical Trials Registry (No. 12621001008819) [9]. Recruitment commenced on 16 August 2021 and will end on 2 October 2021. Data collection will continue until the end of November 2021.

## Data Availability

We will make available upon reasonable request the de-identified individual quantitative participant data collected during the trial and the study protocol after the publication of the trial results.

## Abbreviations

CMNI-30-SR: Conformity to Masculine Norms Inventory 30—Self-Reliance subscale
DSI-SS: Depression Symptom Inventory—Suicidality Subscale
GHSQ-Self: General Help-Seeking Questionnaire—Self
GHSQ-Other: General Help-Seeking Questionnaire—Other
HSUQ: Health Service Use Questionnaire
MDRS-7: Male Depression Risk Scale-7
PLS: Plain Language Statement
RCT: Randomised controlled trial

## References

[1] Slade T, Johnston A, Oakley-Browne M, Andrews G, Whiteford H (2007). National Survey of Mental Health and Wellbeing: methods and key findings. Aust N Z J Psychiatry..

[2] Australian Bureau of Statistics. Causes of death, Australia. 2019. https://www.abs.gov.au/statistics/health/causes-death/causes-death-australia/2019. Accessed 6 May 2021.

[3] Burgess P, Pirkis J, Slade T, Johnston A, Meadows G, Gunn J (2009). Service use for mental health problems: findings from the 2007 National Survey of Mental Health and Wellbeing. Aust N Z J Psychiatry..

[4] Rice S, Fallon B, Bambling M (2011). Men and depression: the impact of masculine role norms throughout the lifespan. Aust Educ Dev Psychol.

[5] Coleman D (2014). Traditional masculinity as a risk factor for suicidal ideation: cross sectional and prospective evidence from a study of young adults. Arch Suicide Res..

[6] Easton S, Renner L, O'Leary P (2013). Suicide attempts among men with histories of child sexual abuse: examining abuse severity, mental health and masculine norms. Child Abuse Negl..

[7] Monk D, Ricciardelli L (2003). Three dimensions of the male gender role as correlates of alcohol and cannabis involvement in young Australian men. Psychol Men Masculinity..

[8] Levant RF, Wimer DJ, Williams CM, Smalley KB, Noronha D (2009). The relationships between masculinity variables, health risk behaviors and attitudes toward seeking psychological help. Int J Men’s Health..

[9] Ftanou M, Cox G, Nicholas A, Spittal MJ, Machlin A, Robinson J, et al. Suicide prevention public service announcements (PSAs): examples from around the world. Health Commun. 2016:1–9. Available from:. 10.1080/10410236.2016.1140269. 10.1080/10410236.2016.1140269 27308843

[10] Pirkis J, Rossetto A, Nicholas A, Ftanou M, Robinson J, Reavley N (2019). Suicide prevention media campaigns: a systematic literature review. Health Commun..

[11] King KESM, Spittal MJ, Phelps A, Pirkis J (2018). Can a documentary increase help-seeking intentions in men? A randomised controlled trial. J Epidemiol Commun Health..

[12] Joiner T, Pfaff JJ, Acres JG (2002). A brief screening tool for suicidal symptoms in adolescents and young adults in general health settings: reliability and validity data from the Australian National General Practice Youth Suicide Prevention Project. Behav Res Ther..

[13] Wilson CJ, Deane FP, Ciarrochi JV, Rickwood D. Measuring help seeking intentions: properties of the general heelp seeking questionnaire; 2005.

[14] Levant RF, McDermott R, Parent MC, Alshabani N, Mahalik JR, Hammer J (2020). Development and evaluation of a new short form of the Conformity to Masculine Norms Inventory (CMNI-30). J Couns Psychol.

[15] Herreen D, Rice S, Zajac I. Brief assessment of male depression in clinical care: Validation of the Male Depression Risk Scale short form in a cross-sectional study of Australian men. BMJ Open. 2022;12(3):e053650. 10.1136/bmjopen-2021-053650. 10.1136/bmjopen-2021-053650 PMC8961138 35351704

[16] Le LK-D, Sanci L, Chatterton ML, Kauer S, Buhagiar K, Mihalopoulos C (2019). The cost-effectiveness of an internet intervention to facilitate mental health help-seeking by young adults: randomized controlled trial. J Med Internet Res..

[17] Independent Housing Pricing Authority (2021). Pricing.

[18] Australian Government Department of Health. Medical Costs Finder. 2021. https://www.health.gov.au/resources/apps-and-tools/medical-costs-finder. Accessed 27 Sept 2021.

